# General Cell-Binding Activity of Intramolecular G-Quadruplexes with Parallel Structure

**DOI:** 10.1371/journal.pone.0062348

**Published:** 2013-04-26

**Authors:** Tianjun Chang, Cui Qi, Jie Meng, Nan Zhang, Tao Bing, Xianda Yang, Zehui Cao, Dihua Shangguan

**Affiliations:** 1 Beijing National Laboratory for Molecular Sciences, Key Laboratory of Analytical Chemistry for Living Biosystems, Institute of Chemistry, Chinese Academy of Sciences, Beijing, China; 2 National Center for Nanoscience and Technology, Beijing, China; 3 Institute of Basic Medical Sciences, Chinese Academy of Medical Sciences & Peking Union Medical College, Beijing, China; International Centre for Genetic Engineering and Biotechnology, Italy

## Abstract

G-quadruplexes (G4s) are four-stranded nucleic acid structures adopted by some repetitive guanine-rich sequences. Putative G-quadruplex-forming sequences (PQSs) are highly prevalent in human genome. Recently some G4s have been reported to have cancer-selective antiproliferative activity. A G4 DNA, AS1411, is currently in phase II clinical trials as an anticancer agent, which is reported to bind tumor cells by targeting surface nucleolin. AS1411 also has been extensively investigated as a target-recognition element for cancer cell specific drug delivery or cancer cell imaging. Here we show that, in addition to AS1411, intramolecular G4s with parallel structure (including PQSs in genes) have general binding activity to many cell lines with different affinity. The binding of these G4s compete with each other, and their targets are certain cellular surface proteins. The tested G4s exhibit enhanced cellular uptake than non-G4 sequences. This uptake may be through the endosome/lysosome pathway, but it is independent of cellular binding of the G4s. The tested G4s also show selective antiproliferative activity that is independent of their cellular binding. Our findings provide new insight into the molecular recognition of G4s by cells; offer new clues for understanding the functions of G4s *in vivo*, and may extend the potential applications of G4s.

## Introduction

G-quadruplexes (G4s) are four-stranded secondary structures of nucleic acids containing repetitive G-rich nucleotides [1]. These structures are stabilized by Hoogsteen hydrogen bonding among four guanine bases and the stacking of planar guanine quartets [2]. Genome-wide bioinformatic searches have revealed that putative G-quadruplex-forming sequences (PQSs) are highly prevalent in human genes, especially in promoter regions of oncogenes and telomere ends [3]–[5]. Accumulating evidence suggests the important role of G4s *in vivo* in regulating gene expression, especially the expression of a number of well-characterized oncogenes, such as c-kit2 [6], RET [7], VEGF [8], c-Myc [9], Bcl-2 [10], and YY1 [11]. Nevertheless, the structures and functions of most PQSs in genome are unknown, suggesting research in this field is still at an early stage [5].

Aptamers are artificial nucleic acid ligands usually generated by SELEX (systematic evolution of ligands by exponential enrichment)[12]. Many reported aptamers adopt G4 structures for target binding [13]–[16]. AS1411 (also known as AGRO100), a G4 DNA aptamer, is currently in phase II clinical trials as an anticancer agent. This molecule is reported to bind cancer cells by targeting nucleolin, a multifunctional protein that is overexpressed by cancer cells, both in the cytoplasm and on the cell surface [17]. Besides as an anticancer agent, this G4 DNA has been extensively investigated as a target-recognition element for cancer-cell-specific drug delivery or cancer cell imaging [18]–[20]. Aside from AS1411, some synthetic G4s have also been reported to exhibit antiproliferative activity against tumor cell lines [21]. However, the molecular basis of the antitumor activity of these sequences remains unclear.

PQSs are also found in aptamers that were selected using whole tumor cells as targets [22]–[24]. A PQS containing aptamer, sgc4, generated against a leukemia cell line CCRF-CEM, is found to bind to many other cell lines [25]. These results led us to presume that G4s in general may be able to bind to many different cells. Additionally, G4 structures are found more stable than other nucleic acid structures in serum or living cells [26], [27], which implies that G4 motifs resulted from the degradation of nucleic acids may be present *in vivo* at a higher level than other forms of nucleic acids. Additional investigation of the interaction between G4s and cells should be of great importance for the discovery and understanding of potential functions of G4s *in vivo*, and may also provide new insight into the molecular mechanisms of the antiproliferative activity of G4s.

In this report, we have investigated the binding pattern of 12 intramolecular G4s to different cell lines, and found that the parallel G4 structure was critical for cell binding. The targets of the G4s were preliminarily determined to be associated with cell surface proteins. Subsequently, the cellular internalization, localization and antiproliferative activity of G4s have also been investigated.

## Materials and Methods

### Materials

All DNAs were synthesized by Sangon Biotech Co. Ltd (Shanghai, China), and purified in our laboratory by HPLC (FL2200, Wenling, China) with a C_18_ column (Agela, 5 µm, 100Å, 4.6× 250 mm, China). The DNA sequences used in this study are listed in Table 1. Unless otherwise indicated, stock solution of DNAs (40 µM) were prepared in Tris-HCl buffer (25 mM, pH7.6) and stored at −20°C. Herring sperm DNA (HS-DNA, <50 bp) was obtained from Sigma-Aldrich Co. LLC. (USA). Antibodies were obtained from the following sources: mouse anti-nucleolin mAb 4E2 (ab13541), mouse IgG1 (ab91353) from Abcam (Cambridge, UK); mouse anti-nucleolin mAb MS-3 (SC-8031, raised against amino acids 1–706 representing full length nucleolin of human origin), PE-conjugated goat anti-mouse IgG1 (SC-3764) from Santa Cruz Biotechnology (California, USA). Lysosome probe (LysoTracker Red, LTR) was purchased from Beyotime Institute of Biotechnology (Shanghai, China). Adriamycin (ADM) was purchased from Meilun Biotech Co. Ltd (Dalian, China), dissolved in sterile water (5 mg/ml), and stored at −20°C. All other reagents were purchased from China National Medicine Corporation Ltd.

**Table 1:** DNA sequences used in this paper.

Name	Sequence (5′-3′)	G4 structure
T18	T_18_	non G4
L30	N_30_	non G4
AS1411	T_2_(TG_2_)_4_T_2_G(TG_2_)_4_ a	mixed parallel/antiparallel
CRO	T_2_(TC_2_)_4_T_2_C(TC_2_)_4_	non G4
TBA	T_3_G_2_T_2_G_2_TGTG_2_T_2_G_2_ a	antiparallel
HT	(T_2_AG_3_)_4_	antiparallel
Oxy28	T_3_G_4_T_4_G_4_T_4_G_4_T_4_G_4_ a	mixed parallel/antiparallel
EAD	T_2_(TG_3_)_4_ a	parallel
Bcl-2	T_3_G_3_CGCG_3_AG_2_A_2_G_5_CG_3_ a	parallel
VEGF	T_3_(G_3_C)_2_CG_5_CG_3_ a	parallel
c-Myc	T_3_GAG_3_TG_4_AG_3_TG_4_A_2_ a	parallel
HIF-1α	T_3_(G_3_A)_2_GAG_5_CG_3_ a	parallel
RET	T_2_A(G_4_C)_3_G_3_ a	parallel
ILPR	T(ACAG_4_TGTG_4_)_2_	mixed parallel/antiparallel
c-Kit2	T_3_(CG_3_)_2_CGCG(AG_3_)_2_ a	parallel

a Because quenching of the fluorophore occurred when it was located adjacent with guanine bases, a spacer consisting of three or two thymidines were added at the 5′end of these sequences. The added spacer did not affect the formation of G4 structure of these sequences.

### Cell Culture

Unless otherwise indicated, cells were routinely grown in a humidified incubator at 37°C/5% CO_2_ and in basal medium supplemented with 10% fetal bovine serum (FBS, Gibcio), and 1% penicillin/streptomycin (Hyclone). HeLa (cervical adenocarcinoma), MCF-7 (hormone-dependent breast cancer), SK-HEP-1 (Human hepatoma), A549 (lung cancer), RAEC (rat endothelial cell of aorta), and HEK293 (embryonic kidney fibroblast) cells were purchased from Cell Resource Center of Shanghai Institute for Biological Sciences (Chinese Academy of Sciences, Shanghai, China), and grown in Dulbecco’s Modified Eagle Medium (DMEM, Gibico). HCT-8 (colon cancer), Jurkat E6-1 (acute T cell leukemia), K562 (leukemia), MRC-5 (normal embryonic lung fibroblast) and MDA-MB-231 (hormone-independent breast cancer) cells were purchased from Cell Culture Center of Institute of Basic Medical Sciences (Chinese Academy of Medical Sciences, Beijing, China), and grown in RPMI 1640 (Gibico) medium except that MDA-MB-231 was grown in L-15 (Hyclone) medium, and cultured without CO_2_. MCF-7/ADM (ADM-resistant MCF-7 subline) cells were purchased from Shanghai Aiyan Biological Technology Co. Ltd (Shanghai, China), grown in RPMI 1640 medium with 1 µg/ml ADM. Before use, MCF-7/ADM cells were seeded in medium without ADM.

### Flow Cytometric Analysis

#### Cells stained by FAM labeled DNAs

All the binding assays are performed on ice. Adherent cells were cultured for 24–48 h, washed with phosphate buffered saline (PBS) to remove the culture medium, then detached by PBS containing 0.02% EDTA buffer (PBS-EDTA, pH 7.4), and washed with ice-cold PBS before use. Suspension cells were washed with PBS before use. The treated cells were incubated with 0.4 µM FAM labeled DNAs (F-DNAs) in 200 µl of binding buffer (PBS with 1 mM CaCl_2_, 5 mM MgCl_2_, 1 mg/ml BSA and 0.1 mg/ml herring sperm DNA) for 45 min.

#### Cells stained by anti-nucleolin antibodies

To measure the nucleolin expression on cell surface, cells were preincubated with binding buffer for 30 min, washed with PBS, and then incubated with anti-nucleolin mAb 4E2 (20 µg/ml) or MS-3 (10 µg/ml) for 45 min. In the negative control experiments, cells were incubated with isotype-matched mouse IgG1 (ab91353, 10 µg/ml). After being washed with ice-cold PBS, cells were further incubated with secondary antibody (PE-conjugated goat anti-mouse IgG1, 1∶50 dilution in binding buffer) for 45 min.

#### Cells double stained by F-DNA and Propidium Iodide (PI)

Cells were incubated with 0.4 µM F-DNA (F-CRO, F-AS1411, F-EAD, F-VEGF or F-c-kit2) and PI (50 µg/ml) in binding buffer for 45 min on ice.

After incubating, these cells were washed with ice-cold PBS, and suspended in binding buffer (500 µl) for flow cytometry analysis on a Beckman Coulter flow cytometer (Cell Lab Quanta™, USA). 10,000 events were collected for each sample. The data were analyzed by WinMDI 2.9 software.

### Proteinase Treatment for Cells

To test whether the targets of G4s are membrane proteins, proteinase treatment experiments were performed in two ways. The first one, cells (5×10^6^) were washed with PBS and then incubated with 0.05% trypsin/0.53 mM EDTA in HBSS at 37°C for 10 min. FBS was then added to quench the proteinase. After washing with binding buffer, the treated cells were used for DNA-binding assay as described in Flow-Cytometric Analysis. The second, cells were firstly stained by 0.4 µM F-G4s, and then incubated with 0.05% trypsin/0.53 mM EDTA in HBSS at 37°C for 10 min, and finally cells were washed and analyzed by flow cytometry.

### Circular Dichroism (CD) Spectra

DNAs (40 µM) were dissolved in Tris-HCl buffer with or without 150 mM NaCl and 10 mM KCl, denatured at 95°C for 10 min, and then cooled slowly to room temperature before use. To mimic the binding of DNAs to cells, all the DNAs were diluted with ice-cold PBS (containing 5 mM MgCl_2_ and 1 mM CaCl_2_) to final concentration of 2–4 µM, and then placed on ice for 45 min before measurement. CD spectra (220–320 nm) was collected at 4°C with a JASCO J-815 CD Spectropolarimeter (JASCO Ltd., Japan) at a rate of 500 nm/min using 400 µl solution in a 1-cm fused quartz cell. To facilitate comparison, the CD spectra were applied background subtraction, and smoothed, so that molar ellipticities could be obtained.

### Unlabled DNAs Competing the Binding of F-DNAs or Antibody

Cells were treated as described above, and incubated with 10 µM unlabeled L30, AS1411, EAD or VEGF in 200 µl binding buffer on ice for 45 min. Then 0.4 µM F-DNAs (F-AS1411, F-EAD and F-VEGF) or mAb MS-3 (10 µg/ml) was added into each sample respectively and incubated for another 45 min on ice. The cells incubated with MS-3 were further incubated with PE-conjugated secondary antibody (1∶50 dilution in binding buffer) for 45 min on ice. Then these cells were washed with ice-cold PBS and resuspended in 500 µl of binding buffer for flow-cytometric assay. Samples without addition of unlabled DNAs were used as controls.

### Measurement of Cellular Internalization of G4s

Cells were seeded in 12-well plates at 4 × 10^5^ cells per well. In the exponential growth phase, F-L30, F-CRO, F-AS1411, F-EAD or F-VEGF was added into culture media with or without FBS at a final concentration of 10 µM and incubated with the cells for 2 h in a humidified 37°C/5% CO_2_ incubator. Cells were treated with trypsin to remove the surface-bound DNAs, washed with ice-cold PBS and then suspended in binding buffer (500 µl) for flow cytometry assay.

### Confocal Microscopy

For binding assay, MCF-7/ADM cells were grown on confocal dishes for 24 h. After washed with ice-cold PBS, the cells were incubated with 0.5 µM F-DNAs on ice for 45 min. Then, the cells were washed with 1 ml of binding buffer and observed under an OLYMPUS FV1000-IX81 confocal microscope (Olympus Corporation, Japan). Confocal images (512 × 512 pixels) were obtained using a 100× objective lens and the images were overlaid using Olympus FV10-ASW 1.6 viewer software.

For internalization studies, the cells were incubated with 10 µM F-DNAs in medium with 10% FBS at 37°C for 2 h, washed with PBS, and then incubated with 50 nM LTR at 37°C for 30 min. They were then washed again with PBS and visualized alive by confocal microscopy.

### Stability Assay of F-DNAs

MCF-7/ADM cells were incubated with 10 µM F-DNAs in medium with 10% FBS at 37°C. The culture media were collected at different time, denatured by 20 mM EDTA and stored at −20°C. The samples containing F-DNAs were analyzed by denaturing-PAGE (12%). Before electrophoresis, DNA samples (10 µM) were denatured at 95°C for 10 min, cooled on ice, and mixed with formamide loading buffer. The gels were exposed to UV light and photographed. The fluorescence intensities (quantified by BioSens software (Shanghai Bio-Tech Co.,Ltd)) of the major bands corresponding to intact DNAs were used to evaluate the stability of the F-DNAs.

### Cell Proliferation Assay

Cell proliferation assay was assessed by Cell Counting Kit-8 (CCK-8, Dojindo Molecular Technologies, Inc.) assay. MCF-7/ADM (5×10^3^ cells per well), Jurkat E6-1 (1×10^4^ cells per well) and K562 (1×10^4^ cells per well) were seeded in 96-well plates, grown for 24 h, then added in triplicate with different concentration of DNAs (CRO, AS1411, EAD, VEGF, and c-kit2) and further grown for 24–120 h. Then CCK-8 reagent (100 µl/ml medium) was applied and incubated with cells at 37°C/5% CO_2_ for 1 h. The cell numbers were measured as the absorbance (450 nm) of reduced WST-8 (2-(2-methoxy-4-nitrophenyl)-3-(4-nitrophenyl)- 5-(2,4-disulfophenyl)-2H-tetrazolium, monosodium salt).

## Results and Discussion

### The Binding Pattern of G4s to Various Cell Lines

In order to study the binding of G4s to different cell lines, we primarily chose ten G4s (Table 1) for testing, including seven PQSs existing in different gene promoters (c-Myc, Bcl-2, VEGF, c-kit2, HIF-1α, RET, and ILPR)[6], [7], [28], [29], one telomere sequence (HT) [30], AS1411 and one G4 aptamer (EAD) [13]. A 30-mer random DNA library, L30 and an 18-mer poly T, T18, were used as controls. All these sequences were labeled with a carboxyfluorescein (FAM) at their 5′ end (F-DNAs) for flow cytometry and confocal imaging assay. As AS1411 has been reported to possess antiproliferative activity against several tumor cell lines [17], nine tumor cell lines and three normal cell lines were chosen for studying effects of G4 binding. To eliminate nonspecific binding, 1 mg ml^−1^ BSA and 0.1 mg ml^−1^ herring sperm (HS) DNA were added to the binding buffer. To avoid cell uptake of these sequences, the binding experiment was performed on ice.

**Figure 1:**
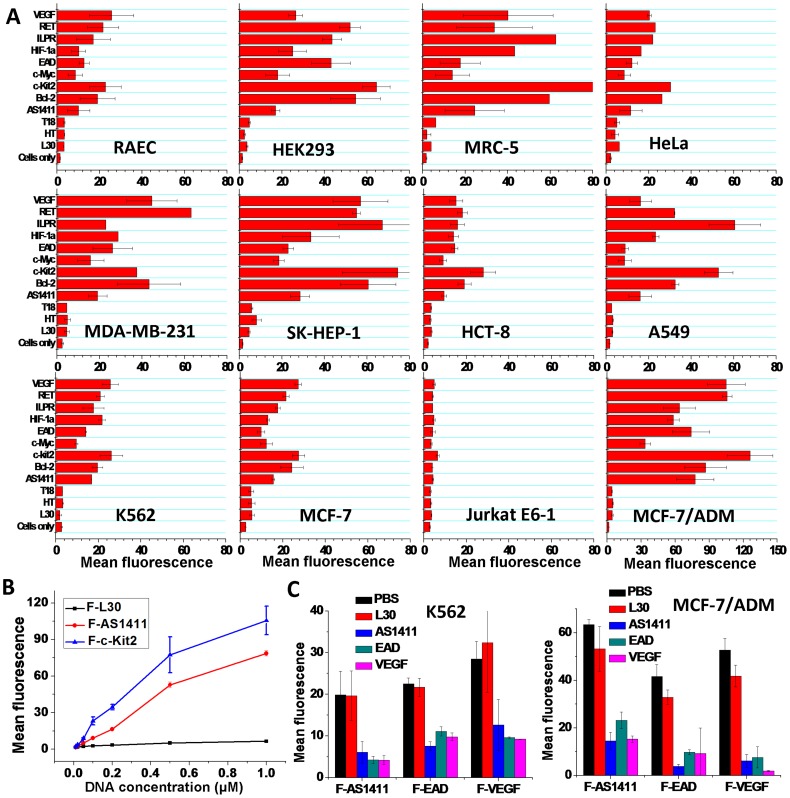
Cellular binding of F-DNAs. **A,** Geometric mean fluorescence of different cell lines stained by F-DNAs (0.4 µM) (n = 2; bars: SEM). **B,** Binding curves of F-L30, F-AS1411 and F-c-kit2 binding to K562 cells. The concentrations of F-DNAs were 0.01, 0.02, 0.05, 0.1, 0.2, 0.5, and 1.0 µM (n = 3; bars: SEM). **C,** The binding of F-AS1411, F-EAD and F-VEGF (0.4 µM) to cells inhibited by unlabeled DNAs (L30, AS1411, EAD, VEGF, 10 µM) (n = 3; bars: SEM). PBS used as control.

The results of flow cytometry assay are summarized in Figure 1. F-L30, F-T18 and F-HT did not show significant binding to any tested cells. Whereas other G4s obviously bound to all the tumor cell lines except for Jurkat E6-1. The flow cytometry assay with Propidium Iodide (PI)/F-DNAs double staining showed that most of the F-DNA stained cells are viable (Figure S1), which excludes the non-specific internalization of G4s by dead cells. The confocal imaging also showed that these G4s bound on the cell surface (Figure S2). It is surprising that three normal cell lines, HEK293, MRC-5 and RAEC were also bound by all G4s except HT. Furthermore, different cell lines showed diverse binding abilities to each G4, and meanwhile different G4s showed different binding abilities to each cell line. A similar binding pattern for all G4s on different cell lines was found. Several G4s showed stronger cellular binding than AS1411. Interestingly, an Adriamycin (ADM) resistant human breast cancer cell subline, MCF-7/ADM showed much higher binding ability (about four-fold higher) to G4s than the ADM-sensitive human breast cancer cell line, MCF-7, suggesting a higher expression of the target molecules of the G4s on MCF-7/ADM.

The binding curves of F-c-kit2 and F-AS1411 to K562 cells within the concentration range of 0.1–1 µM (Figure 1B) indicate an increase in a concentration-dependent manner while F-L30 does not show obvious increase, which further suggests that the G4 binding to cells is based on specific interaction.

The competitive binding assay (Figure 1C) showed that 25-fold excess of unlabeled L30 did not significantly compete with the binding of F-AS1411, F-EAD or F-VEGF to K562 or MCF-7/ADM cells, but the unlabeled AS1411, EAD and VEGF could cross-inhibit the binding of F-G4s by up to 56–90%, suggesting these G4s may bind to the same targets on cell surface.

### The Relationship between G4 Structure and Cellular Binding

Based on the arrangement and orientation of the strands, G4s can be classified into three main conformations, i.e. parallel, anti-parallel and mixed parallel/anti-parallel structures [1]. Circular dichroism (CD) spectra signals can provide information concerning the structure of G-quadruplex: the anti-parallel structure has a positive ellipticity maximum at 295 nm and a negative minimum at 260 nm; the parallel structure has a positive maximum at 264 nm and a negative minimum at 240 nm; and the mixed parallel/anti-parallel structures show a positive maximum at 295 nm plus a positive shoulder near 265 nm and a negative minimum at 235–240 nm [1].

**Figure 2:**
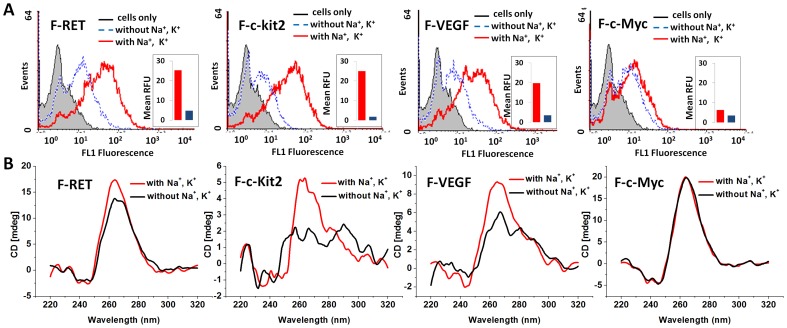
Cellular binding (A) and CD spectra (B) of F-PQSs treated in different buffers. F-PQSs were heat-denatured and annealed in buffers with or without 150 mM Na^+^ and 10 mM K^+^ respectively before dilution for measurement. For flow cytometric assay, final concentration of F-PQSs was 0.4 µM; cell line, K562. The inset histograms represent the geometric mean fluorescence of cells after deducted the auto-fluorescence. For CD spectra measurement, the final concentration of F-RET, F-c-Myc and F-c-Kit2 was 4 µM; F-VEGF was 2.6 µM.

In order to confirm whether the PQSs indeed adopted a G4 structure to bind cells, we compared the binding behaviors and the CD spectral features of the F-DNAs treated in different buffers. It is well known that monovalent cations, such as Na^+^ and K^+^ play an important role in the formation and stabilization of G-quadruplex structures [31]. Therefore, F-RET, F-c-kit2, F-VEGF and F-c-Myc were heat-denatured and then annealed in Tris-HCl buffer with or without 150 mM Na^+^ and 10 mM K^+^. Then the annealed F-DNAs were diluted in PBS (containing 5 mM MgCl_2_ and 1 mM CaCl_2_) for CD measurement and binding assay. As shown in Figure 2A, the cellular binding of F-DNAs treated in buffer without Na^+^/K^+^ was much lower than those treated in Na^+^/K^+^ buffer. The mean fluorescence intensities (after cellular auto-fluorescence deducted) of K562 cells stained by F-DNAs (F-RET, F-c-kit2, F-VEGF and F-c-Myc) treated in buffer with Na^+^/K^+^ were 5.3, 13.7, 5.7, and 1.8-fold higher than those stained by the same DNAs treated in buffer without Na^+^/K^+^. The CD spectra of all the test sequences treated in both solutions showed positive ellipticity maximum at 264 nm (Figure 2B), indicating the parallel G4 structure was adopted by these sequences. However, the positive ellipticities at 264 nm for F-RET, F-c-kit2, F-VEGF and F-c-Myc treated in buffer with Na^+^/K^+^ were 1.3, 2.7, 1.7, and 1.02-fold higher than those without Na^+^/K^+^. The enhanced positive ellipticity indicates higher stability of the G4s treated in Na^+^/K^+^ solution. A good correlation was revealed between the increased fluorescence intensity of K562 cells stained by these sequences and the enhanced positive ellipticity at 264 nm for these sequences. This set of results suggests that the stable G4 structure plays a key role in the binding of G4s on cell surface.

In order to further reveal the relationship between G4 structure and cellular binding, we measured the CD spectra of other DNAs (Figure S3). In the binding buffer, all the cellular binding PQSs showed the CD signals of parallel structure or mixed parallel/anti-parallel structures (ILPR and AS1411); and the non-binding PQS, HT, showed a strong positive ellipticity maximum at 295 nm, which suggests that the anti-parallel G4s could not bind to cells. To confirm this, we further tested the binding behaviors and CD spectra of two reported anti-parallel G4s, TBA (Thrombin binding aptamer) [32] and Oxy28 (Oxytricha nova telomere G4) [33] (Figure S3 and S4). TBA showed the CD signals of anti-parallel G4 structure, and did not show significant binding to cells. In fact, G4s formed from particular PQSs are usually polymorphic in solution [34], parallel and anti-parallel structures can coexist in a particular solution [35], [36]. In the binding buffer, Oxy28 showed the CD signals of the mixed parallel/anti-parallel structures, and weaker cellular binding than AS1411 (Figure S3 and S4). After comparing the positive ellipticities at 260 nm and 295 nm for the G4s with mixed parallel/anti-parallel structures, it was found that G4s with higher ratio of parallel to anti-parallel structures (ratio of ellipticities at 260 to 295 nm) showed higher cellular binding activity (Figure S4). These results suggest that formation of parallel G4 structure is needed for cellular binding of these PQSs.

The above results show the general binding activity of intramolecular parallel G4s to many cell lines, including cancer cell lines, nonmalignant cell lines, adherent cell lines and suspension cell lines. This is the first report on the relationship of G4 structure and cellular binding. This finding provides new insight into the molecular interaction of G4s with different cells, and offers new clues for the selection and design of new cell specific DNA probes. With some of the G4s having stronger cellular binding than AS1411, it offers new clues for the discovery of more potent anticancer G4. Additionally, the strong cellular binding of G4s derived from some genomic promoters may hint at a link between G4s sequences and potential important cellular functions *in vivo.*

### The Targets of G4 Recognition on Cell Surface

Cell membrane contains a variety of biological molecules, such as lipids, proteins and carbohydrates. The proteinase treatment experiment showed that trypsin-treated SK-HEP-1 cells and HeLa cells could no longer bind F-G4s; and the F-G4s binding on untreated SK-HEP-1 cells could be removed by trypsin-treatment (Figure S5). The same results were also observed on MCF-7/ADM cells treated with proteinase K. This set of results suggests that the targets of G4s are membrane proteins or linked to membrane proteins.

**Figure 3:**
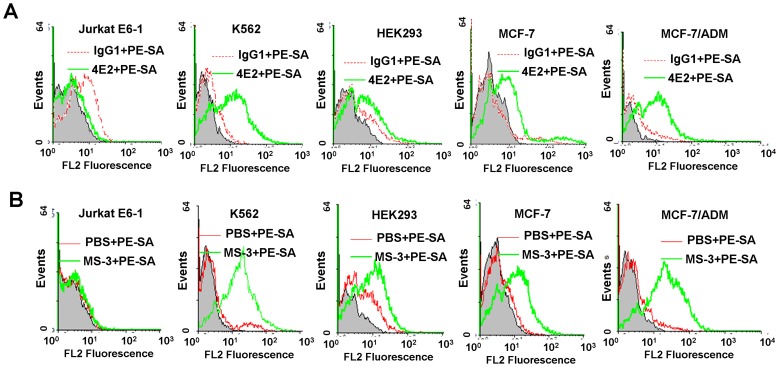
Immunofluorescence assay of anti-nucleolin mAb 4E2 (A) and MS-3 (B) bound to various cell lines. Red curves, IgG1 (negative control); green curves, anti-nucleolin mAb; grey shades, the cellular autofluorescence. PE-SA, PE-conjugated secondary antibody.

The target of AS1411 has been identified to be the cell-surface nucleolin [37]. VEGF and c-Myc have also been reported to bind intracellular nucleolin [37]–[39]. Nucleolin is an abundant, ubiquitously expressed protein that is found in nucleoli, nucleoplasma, cytoplasma and on the surface of some tumor cells [40], [41]. The cell-surface nucleolin was considered to serves as a receptor for proteins, virus and DNA nanoparticles[42]–[44]. In order to investigate whether nucleolin is the target of the tested G4s, the nucleolin expression on cell surface was identified using anti-nucleolin mAb 4E2 (Abcam) and MS-3 (Santa cruz). Nucleolin was found to be highly expressed on MCF-7/ADM and K562 cell lines, moderately expressed on HEK293, MCF-7 cell lines, and very lowly expressed on Jurkat E6-1 cell line (Figure 3), which is consistent with the patterns of G4 binding to these cells. Furthermore, preincubation of cells with 10 µM AS1411, EAD and VEGF reduced binding of anti-nucleolin mAb MS-3 (10 µg/ml) to MCF-7/ADM cells by 76%, 73%, and 77% respectively, while 10 µM unlabeled L30 reduced MS-3 binding by only 39% (Figure S6). These results may suggest that cell-surface nucleolin is one of the targets of these G4s.

### Cellular Selective Uptake of G4s

Several synthetic G4s have been reported to exhibit antiproliferative activity against tumor cell lines [21]. Different mechanisms have been proposed for the internalization of AS1411, including nucleolin mediated endocytosis [45], macropinocytosis and other unknown mechanisms [46]. Since our results have demonstrated that intramolecular parallel G4s have a general binding capability to cells, it would be interesting to find out if this binding could result in cellular uptake.

**Figure 4:**
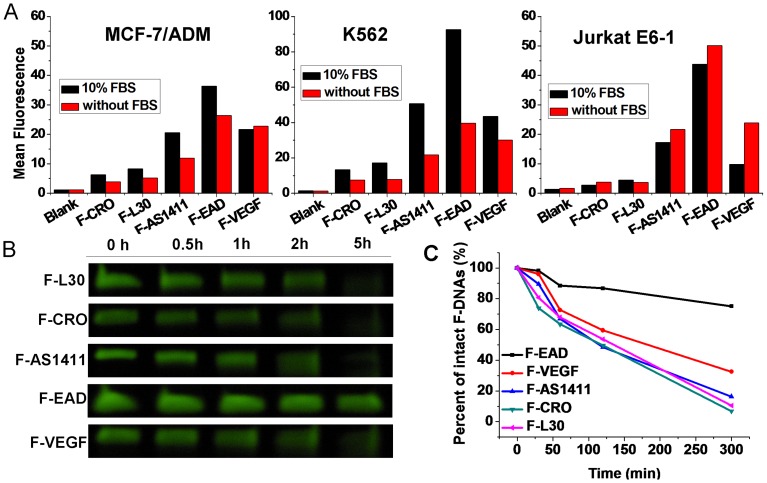
Cellular uptake and Stability of G4s. (A) Uptake of F-DNAs (10 µM) by different cells. Cells were incubated with F-DNAs in media with or without 10% FBS. The geometric mean fluorescence was extracted from flow cytometric results. (B and C) Stability of F-DNAs (10 µM) in medium with 10% FBS during cell culture. Data were normalized to the F-DNAs bands at 0 h.

To investigate the cell uptake of G4s, K562, MCF-7/ADM and Jurkat E6-1 cells were incubated respectively with 10 µM of F-AS1411, F-EAD, F-VEGF, F-L30 and F-CRO (a C-rich non-G4 sequence) [37] in culture media with or without 10% FBS at 37°C for 2 h [46], then treated with trypsin to remove the surface-bound F-DNAs. Finally, the fluorescence inside the cells was measured by flow cytometry assay. As shown in Figure 4A, the uptake of G4s by the tested cells was much higher than that of the control DNAs (F-CRO and F-L30), which is consistent with the observation by Bates’ group that uptake of AS1411 is much more efficiency than non-G4 sequences [46]. The uptake of F-EAD was the highest among the G4s. The presence of 10% FBS in medium was found to help the internalization of G4s to K562 and MCF-7/ADM cells, but not with Jurkat E6-1 cells (Figure 4A). Interestingly, results earlier in this report have shown that G4s did not bind to Jurkat E6-1 cells but strongly bound to MCF-7/ADM cells. These results indicate the uptake of G4s by Jurkat E6-1 cells is likely independent of binding of G4s to cell membrane, which agrees with the observation by Bates’ group [46]. Therefore, the uptake of G4s by Jurkat E6-1 cells may take a different pathway (such as macropinocytic pathway) other than receptor mediated internalization as reported by Fernandes’ group [45].

It is well known that natural oligonucleotides are sensitive to a variety of nucleases commonly present in biological fluids, but G4s have been shown to have increased nuclease resistance [21]. In order to assess the stability of tested F-G4s during cell culture, the culture media containing F-DNAs were collected and analyzed by denaturing PAGE. As shown in Figure 4B and C, after 5-hour incubation with MCF-7/ADM cells in the medium containing 10% FBS, about 75% of F-EAD kept intact, while 68% of F-VEGF was degraded, and other F-DNAs were completely degraded. Even after 48 hour incubation in PBS containing 10% FBS, the intact F-EAD still could be observed (Figure S7). This result suggests that this great stability of F-EAD during the cell culture process might have accounted for its high cellular uptake.

**Figure 5:**
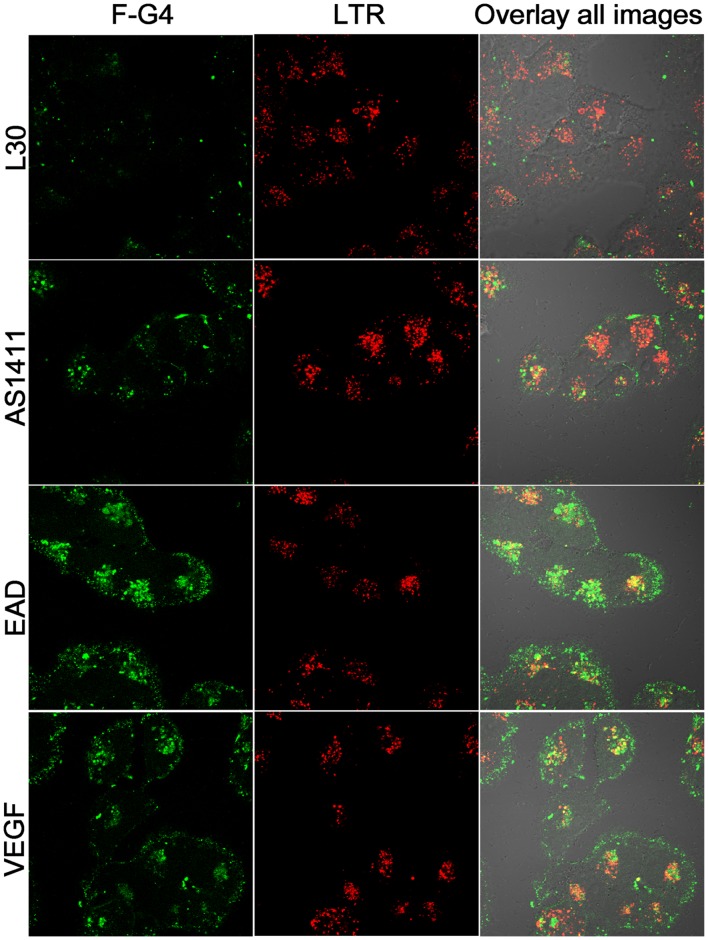
Confocal images of F-G4s and LTR uptaked by MCF-7/ADM cells.

The location of F-G4s in MCF-7/ADM cells was observed by confocal imaging. Very recently, Bates’ group reported that AS1411 was taken into cancer cells by a macropinocytic pathway [46]. Therefore a lysosomal or endosomal marker, LysoTracker Red (LTR), was used as a reference. The confocal images (Figure 5) show that strong fluorescence from F-G4s was observed inside the cells as well as on the cell surface, whereas only very weak fluorescence from F-L30 was observed in cells. The cells incubated with F-EAD showed the strongest fluorescence. These results were consistent with those of the flow cytometry assay. The colocalization of LTR and G4s indicates that the internalized G4s resided in lysosome of MCF-7/ADM cells, and therefore the internalization might be through the endosome/lysosome pathway.

### Selective Antiproliferative Activity of G4s against Cancer Cells

**Figure 6:**
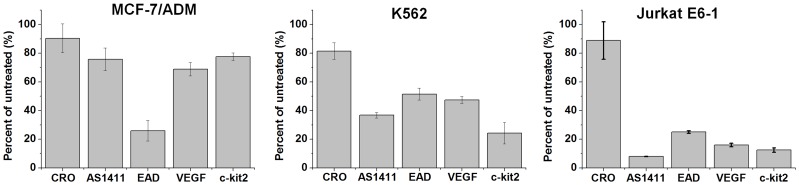
Antiproliferative activity of G4s. DNAs (10 µM) were cultured with cells for 72 h. Cell viability was calculated by taking untreated cells to be 100%, and the background corresponding to medium alone (no cells) was subtracted. n = 3, SEM.

Given that AS1411 and some synthetic G4s were reported to exhibit antiproliferative activity against tumor cell lines [21], the antiproliferative activity of current G4s to MCF-7/ADM, K562 and Jurkat E6-1 cells were measured. As shown in Figure 6, after incubation with MCF-7/ADM, K562 or Jurkat E6-1 cells for 72 h, EAD, VEGF, AS1411 and c-kit2 showed significant antiproliferative activity compared with the control sequence (CRO). EAD showed the highest antiproliferative activity for MCF-7/ADM cells (about 75% mortality); and AS1411 showed the highest antiproliferative activity for Jurkat E6-1 cells (about 90% mortality). And treatment of these three cell lines with different concentration of G4s for 24 to 120 hours caused a dose- and time-dependent decrease in cell proliferation (Figure S8). These results suggest that some G4s possess selective antiproliferative activity. Given that G4s strongly bound to MCF-7/ADM cells but did not bind to Jurkat E6-1 cells, this set of results suggests that the growth inhibition by G4s may be independent of their cellular binding, but rather related to the internalization of G4s. Because nucleolin was found expressed on MCF-7/ADM but not Jurkat E6-1 cells, the surface nucleolin may not be as important in the antiproliferative activity of the G4s. Our results further confirm that intramolecular G4s possess general antiproliferative activity. As PQSs are prevalent in human genes, to discover the unknown mechanism of antiproliferative activity of G4s will lead to insights into basic cell biology, as well as direct the design and optimization of G4 anticancer agents.

### Conclusion

In summary, our investigation found that intramolecular G4s with parallel structure, including some PQSs in genes, generally had the binding ability to many different cell lines. The targets of these G4s were preliminarily identified to be membrane proteins, and the surface nucleolin is one of targets of them. Intramolecular G4s could be selectively taken in by cells, but the uptake is independent of the cellular binding of G4s. These G4s also showed selective antiproliferative activities that is independent of their cellular binding. These results provide new insight into the molecular recognition of G4s by cells, offer new clues for understanding the functions of G4s *in vivo*, and would be of benefit to the design and optimization of G4 anticancer agents.

## Supporting Information

Figure S1 Flow cytometric assay of K562 cells double stained by F-DNA and PI. Cells were incubated with F-DNA (0.4 µM F-CRO, F-AS1411, F-EAD, F-VEGF and F-cikit2) and PI (50 µg/ml) for 45 min on ice, and then analyzed by flow cytometry. Cells stained with PI only were used as control (indicated by “Cells”).
(TIF)

Figure S2 Confocal images of F-L30, F-AS1411, F-EAD, and F-VEGF binding to MCF-7/ADM cells at 4°C.
(TIF)

Figure S3 CD spectra of F-DNAs. The F-DNA stock solution (40 µM) which was used in cellular binding assay was diluted in PBS to a final concentration of 4 µM, and then the CD spectra were measured at room temperature.
(TIF)

Figure S4 Binding of F-TBA (anti-parallel), F-HT (anti-parallel G4) and F-Oxy28 (mixed parallel/anti-parallel G4) to various cell lines.
(TIF)

Figure S5 Investigation of the cellular surface target of G4s. A and B, Binding of F-AS1411, F-EAD and F-VEGF to SK-Hep-1(A) and HeLa (B) cells that were detached by PBS-EDTA or trypsin. C, The fluorescent change of F-G4s stained SK-Hep-1 cells after treated by trypsin.
(TIF)

Figure S6 Inhibition of mAb MS-3 (10 µg/ml) binding to MF-7/ADM cells by 10 µM unlabled DNAs. PE-SA, PE-conjugated secondary antibody. The inset histograms represent the geometric mean fluorescence of cells after deducting auto-fluorescence; blue column: MS-3 binding to cells, green column: MS-3 binding to cells after inhibited by unlabeled DNAs.
(TIF)

Figure S7 Stability of 10 µM F-DNAs (F-L30, F-AS1411 and F-EAD) in 10% FBS. The stability assays of F-DNAs for 72 h was performed in PBS with 10% FBS at 37°C. F-DNAs were collected at 0, 2, 24, 48, and 72 h respectively, and treated as described in main text and analyzed by denatureing-PAGE (12%)
(TIF)

Figure S8 Antiproliferative activities of G4s on different cell lines in different times. Antiproliferative activities of 5, 10, 15 µM G4s to MCF-7/ADM cells (A), K562 cells (B), and 1, 5, 10 µM G4s to Jurkat E6-1 (C).
(TIF)
