# Supplementary figures and images for: General Cell-Binding Activity of Intramolecular G-Quadruplexes with Parallel Structure
Source: PLoS One. 2013 Apr 26;8(4):e62348. doi: 10.1371/journal.pone.0062348 (PMC3637168; doi:10.1371/journal.pone.0062348)

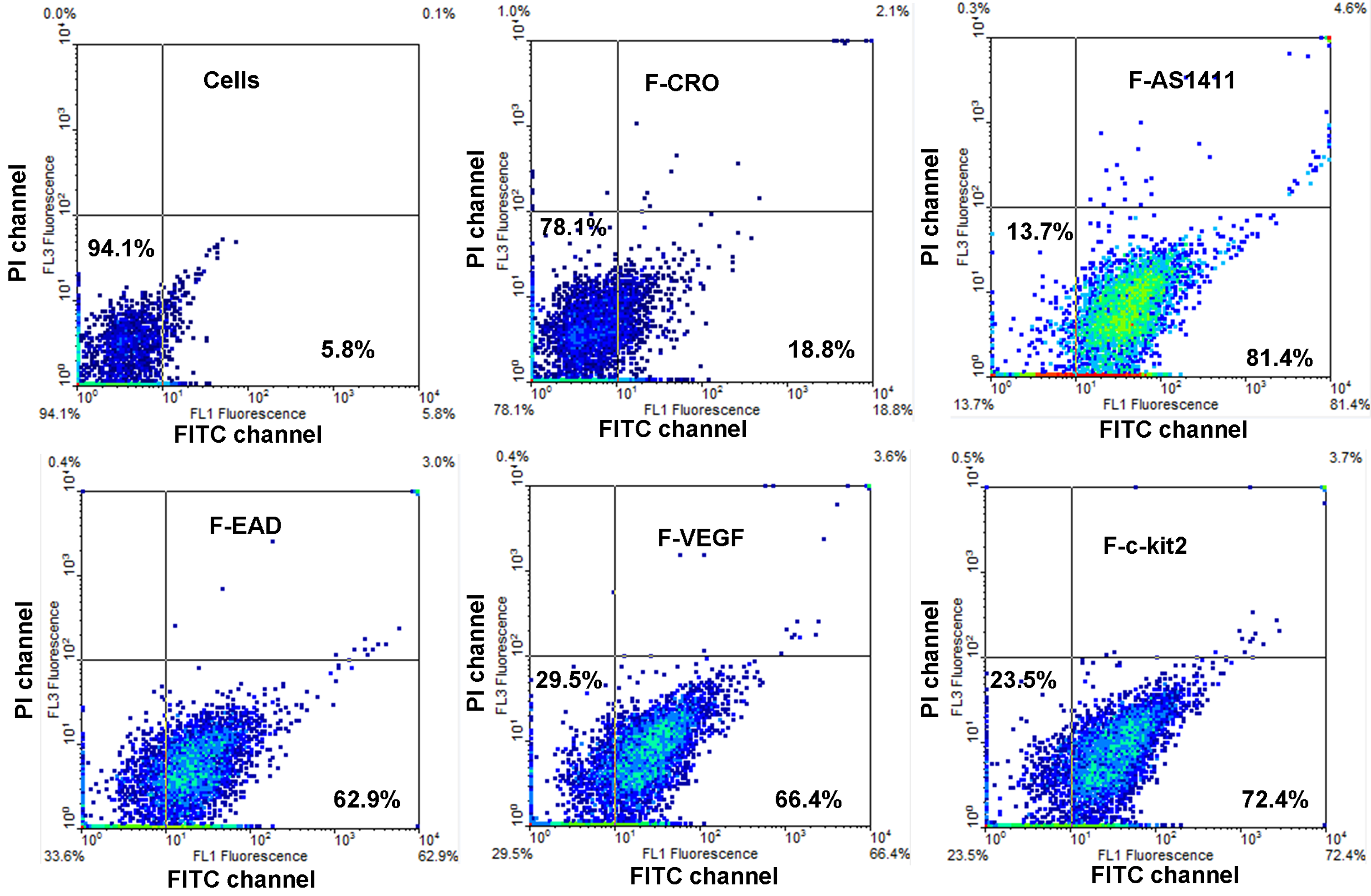

Supplement: Figure S1 — Flow cytometric assay of K562 cells double stained by F-DNA and PI. Cells were incubated with F-DNA (0.4 µM F-CRO, F-AS1411, F-EAD, F-VEGF and F-cikit2) and PI (50 µg/ml) for 45 min on ice, and then analyzed by flow cytometry. Cells stained with PI only were used as control (indicated by “Cells”). (TIF) [file pone.0062348.s001.tif]

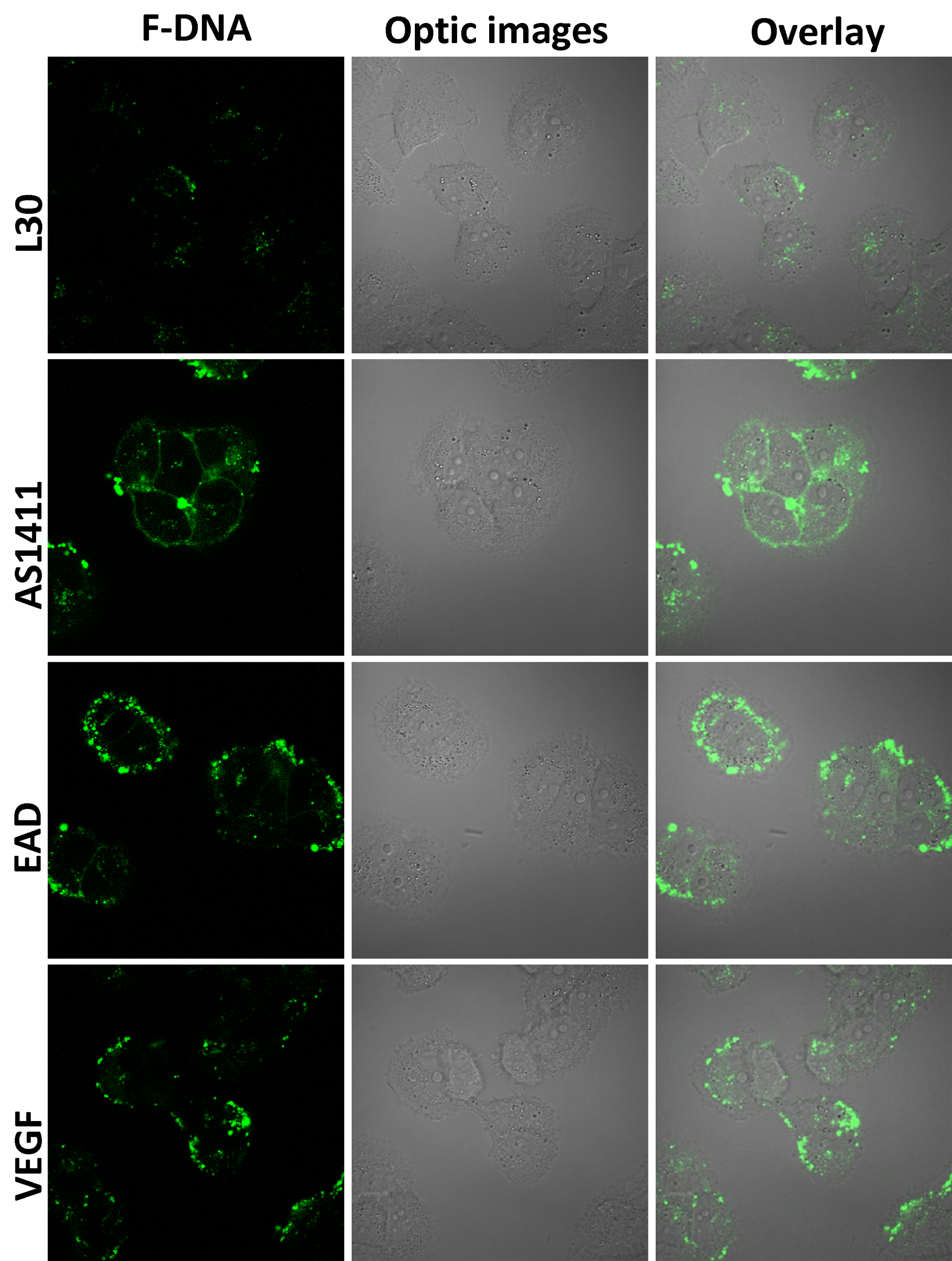

Supplement: Figure S2 — Confocal images of F-L30, F-AS1411, F-EAD, and F-VEGF binding to MCF-7/ADM cells at 4°C. (TIF) [file pone.0062348.s002.tif]

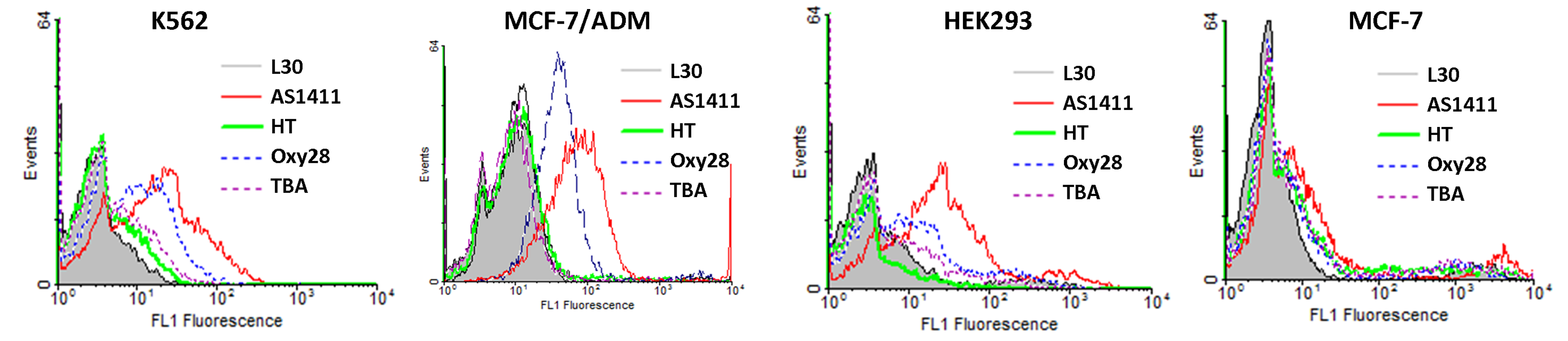

Supplement: Figure S4 — Binding of F-TBA (anti-parallel), F-HT (anti-parallel G4) and F-Oxy28 (mixed parallel/anti-parallel G4) to various cell lines. (TIF) [file pone.0062348.s004.tif]

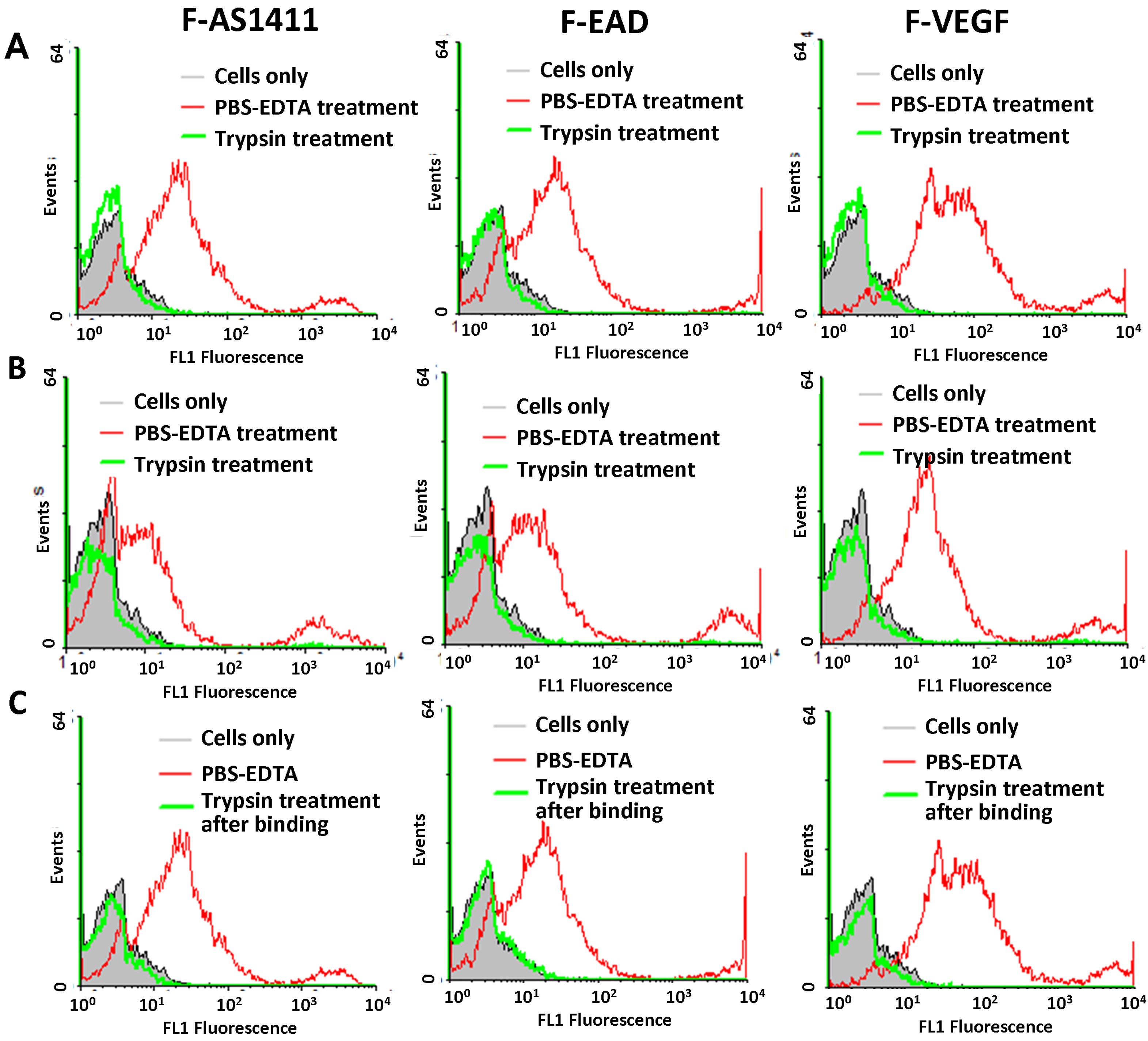

Supplement: Figure S5 — Investigation of the cellular surface target of G4s. A and B, Binding of F-AS1411, F-EAD and F-VEGF to SK-Hep-1(A) and HeLa (B) cells that were detached by PBS-EDTA or trypsin. C, The fluorescent change of F-G4s stained SK-Hep-1 cells after treated by trypsin. (TIF) [file pone.0062348.s005.tif]

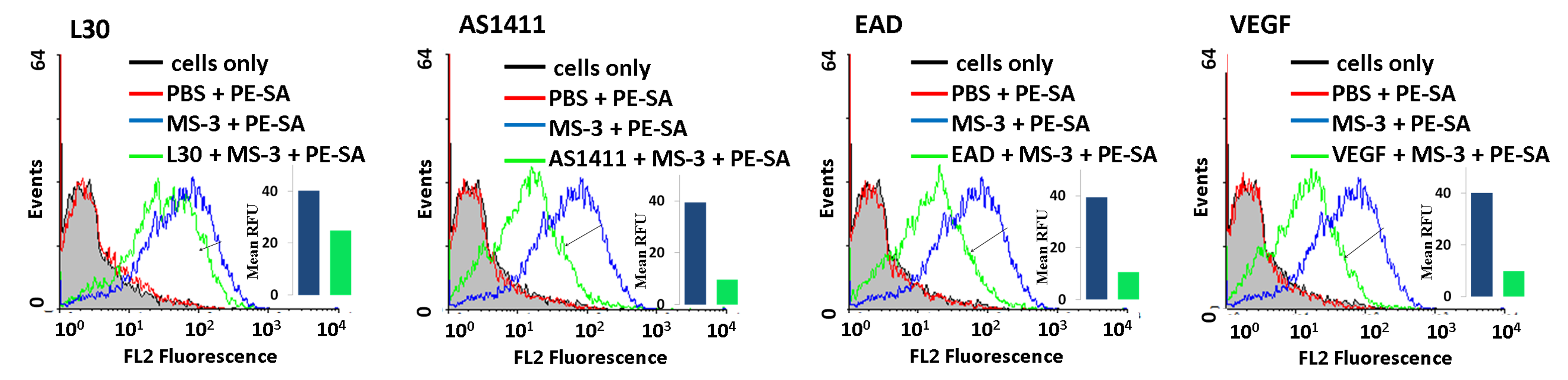

Supplement: Figure S6 — Inhibition of mAb MS-3 (10 µg/ml) binding to MF-7/ADM cells by 10 µM unlabled DNAs. PE-SA, PE-conjugated secondary antibody. The inset histograms represent the geometric mean fluorescence of cells after deducting auto-fluorescence; blue column: MS-3 binding to cells, green column: MS-3 binding to cells after inhibited by unlabeled DNAs. (TIF) [file pone.0062348.s006.tif]

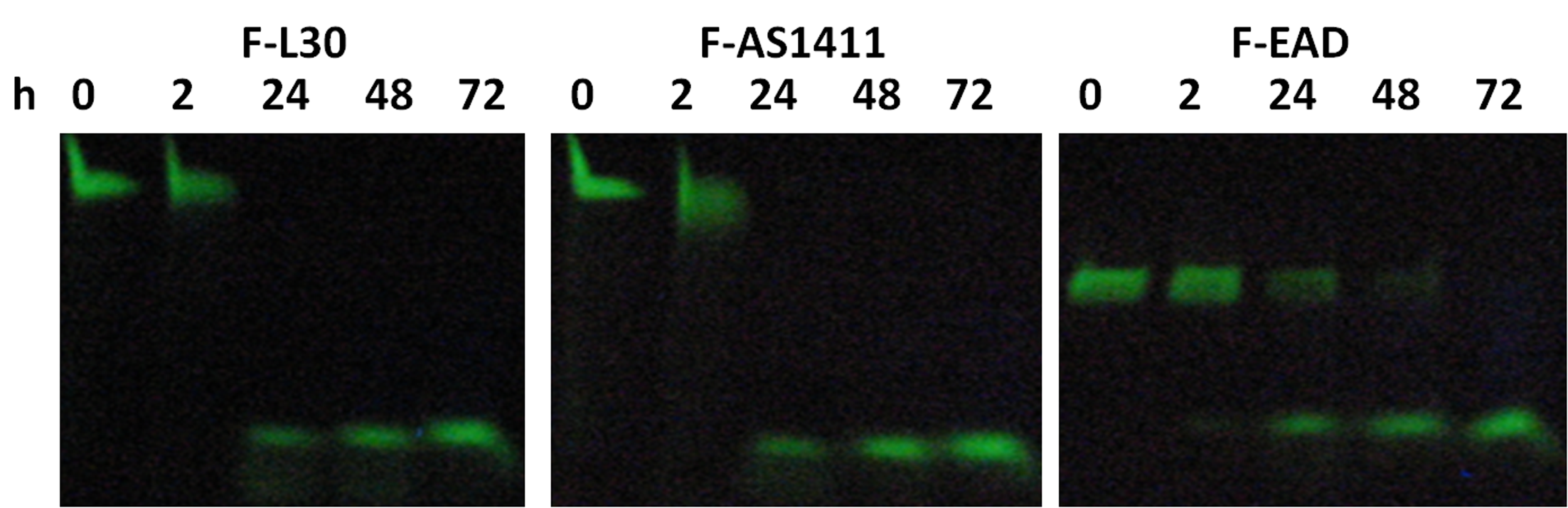

Supplement: Figure S7 — Stability of 10 µM F-DNAs (F-L30, F-AS1411 and F-EAD) in 10% FBS. The stability assays of F-DNAs for 72 h was performed in PBS with 10% FBS at 37°C. F-DNAs were collected at 0, 2, 24, 48, and 72 h respectively, and treated as described in main text and analyzed by denatureing-PAGE (12%) (TIF) [file pone.0062348.s007.tif]

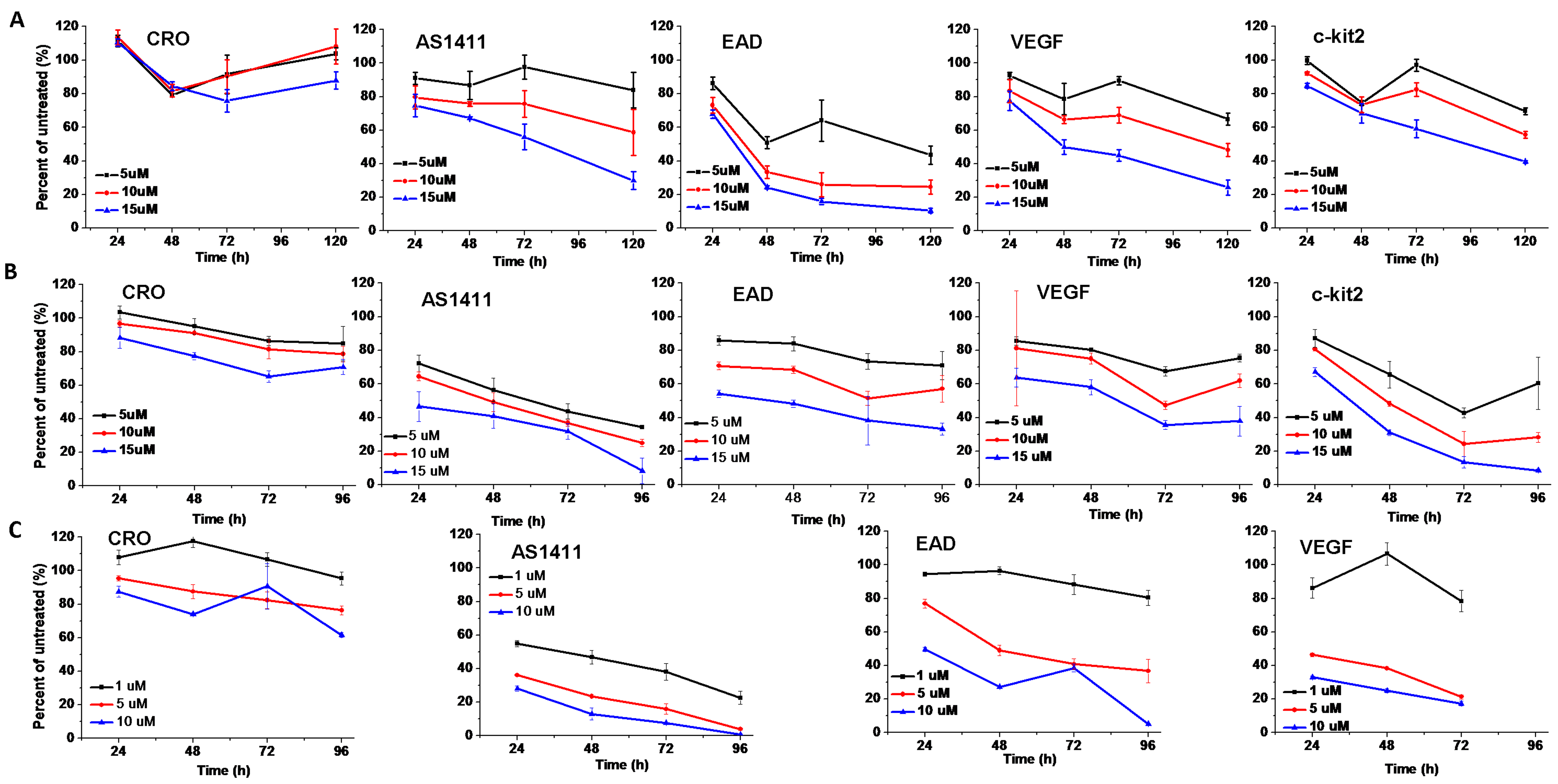

Supplement: Figure S8 — Antiproliferative activities of G4s on different cell lines in different times. Antiproliferative activities of 5, 10, 15 µM G4s to MCF-7/ADM cells (A), K562 cells (B), and 1, 5, 10 µM G4s to Jurkat E6-1 (C). (TIF) [file pone.0062348.s008.tif]
